# Application of Machine Learning Techniques to Analyze Patient Returns to the Emergency Department

**DOI:** 10.3390/jpm10030081

**Published:** 2020-08-07

**Authors:** Antonio Sarasa Cabezuelo

**Affiliations:** Department of Computer Systems and Computing, School of Computer Science, Complutensian University of Madrid, 28040 Madrid, Spain; asarasa@ucm.es

**Keywords:** machine learning algorithms, neural networks, emergency medicine

## Abstract

The study of the quality of hospital emergency services is based on analyzing a set of indicators such as the average time of first medical attention, the average time spent in the emergency department, degree of completion of the medical report and others. In this paper, an analysis is presented of one of the quality indicators: the rate of return of patients to the emergency service less than 72 h from their discharge. The objective of the analysis was to know the variables that influence the rate of return and which prediction model is the best. In order to do this, the data of the activity of the emergency service of a hospital of a reference population of 290,000 inhabitants were analyzed, and prediction models were created for the binary objective variable (rate of return to emergencies) using the logistic regression techniques, neural networks, random forest, gradient boosting and assembly models. Each of the models was analyzed and the result shows that the best model is achieved through a neural network with activation function tanh, algorithm levmar and three nodes in the hidden layer. This model obtains the lowest mean squared error (MSE) and the best area under the curve (AUC) with respect to the rest of the models used.

## 1. Introduction

The study of the quality system of hospitals is a necessary activity for the improvement of the services they offer [1]. In recent years, different public and private institutions have established a set of activity and quality indicators that allow the evaluation, monitoring and comparison of the activities of hospital emergency services [2]. For example, the Spanish Society of Emergency Medicine developed a minimum set of indicators establishing a common, homogeneous and reliable system of information in the emergency services [3]. Three groups of indicators [4] are defined: activity (they measure the number of requests for assistance), quality (they measure qualitative aspects of the operation of the service) and result (they measure results reporting the quality, technical and decisive capacity). Among the quality indicators are the following [5]: average time of first medical attention [6], average time spent in the emergency department, degree of completion of the history, information to patients and relatives, diagnostic coding of discharges, proportion of admissions, rate of return to the emergency room and mortality rate in the emergency room [3]. This work will analyze one of the quality indicators mentioned: the rate of return to the emergency room [7]. This measures the number of patients who, after being treated in an emergency department and discharged, return in less than 72 h [8]. The rate of return to the emergency room is calculated as the quotient (multiplied by a thousand) between the number of patients who return in less than 72 h and the total number of patients who come to the same service in a given period of time. The importance of the study of re-admissions lies in improving the quality of hospital emergency services [2], since avoiding the patient’s return to the emergency room is a consequence of the quality provided [9]. In the field of private healthcare, it is also included as an index of healthcare quality [10].

There are different approaches to analyze the return to the emergency department that vary according to the factors [11,12] that are taken into account to analyze this indicator. A key factor is the time [13,14] that must elapse from discharge to the next visit to consider said return as a return. In [15] the profile of the patients who return is studied through variables such as age, sex, day of the week or pathology, among others. For this, three different times are used, studying separately the patients who return in the first 24 h, those who return between 24 and 48 h, and those who take up to 72 h. The result showed that 88.76% of the patients who return do so in the first 48 h, so they consider that it is not necessary to use a longer time. On the other hand, [16] analyzes whether the time to return to the emergency room influences the short-term mortality of the patients. From the study carried out, it is defined that the return time is any visit that occurs up to eight days after discharge. In [17,18], the optimal time is considered for re-entry. For this, the data on the return to the emergency room of patients in adulthood over a period of 30 days were analyzed, and it was concluded that the most inclusive time was nine days.

Another factor to analyze this variable is the causes that influence returns [19]. Returns to the emergency room occur when, after a certain time from discharge, the patient returns unscheduled and for the same reason as the first appointment [20]. However, a difficulty that arises in these studies is knowing if the return is due to the same reason for the first visit or is due to a cause derived from the first visit [21]. In general, it is preferable to study the return due to any cause [12]. In [22], a study of the rate of return to the emergency department was carried out in order to search for the factors associated with said return. To do this, they classified the causes into four groups: related to the patient, the doctor, the health system and the disease; a classification that serves as a reference for subsequent work. However, it is a very expensive process [23] since different doctors evaluate each case separately and if there is a discrepancy, it should be evaluated by a committee with several reviewers and a doctor. There are quite a few studies [24] showing that the cause of greatest return is that of the disease or the patient. For example, [25] shows that unscheduled returns that occur in the first week after discharge are due to the disease with 48% and the patient with 41%. [26] studied unscheduled returns in the 72 h from discharge and showed that in 60.4% the first cause of return is due to the disease and the second cause with 20.0% is due to the doctor.

Other studies [27,28,29] analyzed the diagnoses of those patients who return, with the aim of observing if there is any type of relationship between readmission and the disease for which they go to the emergency department. For example, in [30] it was found that renal colic and spondylosis were the most frequent diseases of those individuals who returned, followed by headaches.

In general, the studies carried out [31] are based on the use of descriptive methods on demographic variables (degree of disability or life situation) or quantitative variables (drug count, time markers, or diagnostic codes). In machine learning, [32] random forests and gradient boosting have been used to predict return within 30 days [33], or logical regression [34] for time intervals shorter than 72 h, such as in [35]. Another study [36] used a gradient boosting over a range of 72 h to nine days to analyze data from electronic clinical records [37] such as administrative data (demographics, previous hospital use, comorbidity categories, historical vital values and current), treatment data (laboratory values, ECG and imaging counts, drugs administered), data available at the time of triage and data available at the time of discharge.

This article describes a study on the phenomenon of the return of patients to the emergency department of a hospital in less than 72 h. Firstly, the time limit of 72 h was used as it is the most accepted in the scientific literature and contains a greater number of case studies in the dataset used. On the other hand, the objective of the study was established to find the best set of variables that explain the phenomenon and the best machine learning algorithm to model this phenomenon. For this, the binary variable of the rate of return to the hospital emergency department was considered as the objective study variable. In addition, to carry out the analysis the use of machine learning algorithms was proposed (logistic regression [38], neural networks [39], random forest [40], gradient boosting [41] and assembly models [42]). Many of the studies carried out (discussed in the previous paragraphs) are based on classical statistical techniques. However, in this case, the characteristics of the dataset used (which contains a large amount of data (143,803 observations) from a sufficiently broad period from June 2015 to February 2018 inclusively for a total of 33 months) are ideal to be used with machine learning algorithms. The main contributions of this work are the model and the variables obtained that better explain the phenome analyzed. In this sense, the analysis performed indicates that the best model is a neural network with activation function tanh, algorithm levmar and three nodes in the hidden layer, and the set of variables that best explain the phenomenon are pathology2 (corresponding to general medicine), reason_discharge2 (hospitalization of the plant) and reason_discharge5 (evasion). The model found shows a better behavior than the rest of the studied models (since it presents a lower mean squared error (MSE) than the rest of the models and a better area under the curve (AUC)). In addition, the result is consistent with what is stated in the studies of other authors about the non-linear nature of the phenomenon studied (since neural networks generally model quite well phenomena that have non-linear behavior).

The structure of the paper is as follows. In the first section, we will describe the dataset that has been used. The following explains how the data was prepared before performing the analysis (detection of extreme points, treatment of missing data, transformation of data and selection of variables). The next section describes the results of the analysis carried out with each type of machine learning algorithm. In the discussion section, the results are analyzed together to obtain a response to the stated objectives. Finally, a set of conclusions and lines of future work are proposed.

## 2. Data Analysis

The data analyzed comes from a reference hospital with a population of around 290,000 inhabitants for the period between June 2015 and February 2018 (both included) for a total of 33 months. The number of emergencies attended at this period was 143,803 with a return rate of:

(1) $$Return\;rate=\left(\frac{Number\;of\;returning\;patients}{Total\;number\;of\;patiens}\right)*1000=\left(\frac{6209}{143803}\right)*1000=43.18$$

This means that 4.32% of patients returned to the emergency department in less than 72 h.

The main characteristics of the data are:

- It was anonymous but some personal and medical characteristics of the patients appeared.
- The data were included in each patient’s electronic medical record by the doctors.
- There were 62 variables in the data, where 27 were interval variables, 33 were categorical and 2 were nominal free-field variables.
- The objective variable (which indicates whether a patient returns within 72 h from discharge) was categorical with levels of 0, 1, 2, 3, 4, 5 and 6, where 0 meant that the patient did not return to the emergency department and the rest of the numbers mean that they returned for different reasons.
- There were categorical variables coded in many different ways. In some of cases, it was not possible to determine the number of levels.

Appendix A shows the tables of the variables of the dataset.

## 3. Methodology

This section describes the steps to perform the data analysis. The first subsection shows the data preparation process and second subsection introduces the methods used to analyze the data.

### 3.1. Data Preparation

This subsection describes the stages of data preparation. First, the variables rejected in the analysis are described. Next, a descriptive analysis in order to find anomalies in the data is described. Next, the treatment of the missing data and the transformations of some variables are described. Finally, the selection of the variables that have been considered for study are described.

#### 3.1.1. Variables Rejected

Some variables were rejected for use in the analysis for several reasons that depended on the type of variable:

Interval variables: variables whose content was not interesting for the study were rejected.

- Categorical variables: only those variables that had well-coded levels (no repeated levels with different names) and that did not exceed 25 levels (the variables that exceeded this limit had too many levels to be grouped) were considered.

As result, 24 variables were rejected:

- The nominal free field variables: comment and clinical_judgment were rejected since they did not provide information (they cannot be coded and therefore analyzed).
- The categorical variables: diagnostic_main, entity, diagnostic_group, doctor_family, first_doctor_assigned, first_doctor_consultation, location, doctor_discharge, nhc, procedures, processes and reason_consultation were rejected since they had 25 established and defined levels
- The interval variables: registration_date, registration_medical_date, consultation_date, emergency_date, admission_date, first_date_consultation, first_date_sol_lab, first_date_sol_rad were rejected because they did not provide relevant information to the study.
- The interval variable reconsultation_last_year was rejected because it was miscalculated (hospital members reported this situation)

Therefore, 38 variables remained to carry out the analysis.

#### 3.1.2. Descriptive Analysis

A descriptive analysis was carried out in order to observe the available data and do some modification if necessary. The calculated information depends on the type of variable. The interval variables are described in Table 1 (name of variable, mean, missing data, total data, minimum, maximum, standard deviation, skewness and kurtosis).

The categorical variables are described in Table 2 (name of variable, type variable (C, character and N, Nominal), number of levels and number of missing data).

After the initial exploration, some modifications were done:

- Interval variables: the values that were out of range were modified (Table 3)
- Categorical variables: some levels were grouped (variables with a large number of levels; variables with empty categories that did not represent an absent value and variables with similar classes). The Table 4 shows the modifications.

Other categorical variables were transformed by modifying their scale. The Table 5 shows the modifications.

#### 3.1.3. Elimination of Outliers

Next, the presence of outliers in the interval variables was analyzed. Values that exceeded three standard deviations from the mean for variables with symmetric distributions and nine MADs (median of absolute distances to the median) for variables with asymmetric distributions (the rest) were considered outliers. Data considered outliers were converted to missing values. The variables with outliers were: age, cardiac_frequency, press_arterial_min, discharge_min_time, observation_min_time, and triage_min_time.

#### 3.1.4. Missing Data Treatment

In this phase, the presence of missing data was analyzed. Variables with more than 50% of missing data are eliminated (imputing so many observations will lead to an error) and when the presence of missing values is not very high then the missing data is replaced by valid values (imputation). In this last case, all variables were imputed randomly, taking into account the distribution of each variable. Table 6 shows the variables with missing values: name of variable, number of missing values and decision about its elimination.

#### 3.1.5. Transformation of Variables

In this phase, the variables that need to be transformed were analyzed. Interval variables were not transformed since the tested transformations (logarithm, root, square, and others) did not improve their R squared. However, categorical variables were modified by converting them into dummy variables (as many dichotomous variables created as the number of categories of each original variable). Variables that only had two classes were excluded from this modification.

#### 3.1.6. Selection of Study Variables

As result of the previous phases, there were two datasets: imputed and with missing values. The selection of variables was done separately for the two datasets since there were different values that could impact the performance of the analysis. The variable selection methods used were: R-square, partial least squares, “step-by-step” regression logistic and decision tree. Table 7 shows a selection of imputed data (the dummies variables are represented in the format: variableNumber. For example, pathology2).

Table 8 shows a selection of missing data.

Among the variables selected three sets were defined (Table 9): set A had fewer variables and it was more conservative and robust; set B had more variables and tended to overfit; and set C included all the variables (including the variables of sets A and B) that resulted from the data preparation phases: six interval variables, 42 binary variables (most were dummy variables), one binary target variable, and one variable nominal ID (this variable allows to identify each data). The three sets were tested for each analysis technique in such a way that the one that worked best in each situation was chosen according to the results obtained.

### 3.2. Methods of Evaluation

The dataset was randomly divided into training data (with 70% of the total) to build the model and test data (with 30% of the total) to evaluate the errors. In order to evaluate, the models used the following metrics:

- Misclassification rate: the quotient between the number of erroneous classifications by the technique in the validation set and the total number of observations in the validation.
- AIC (Akaike information criterion): model comparison measure that rewards goodness of fit and penalizes the number of estimated parameters. It is a measure about goodness of fit of the model
- SBC (Schwarz Bayesian criterion): model comparison measure that increases the greater the unexplained variation in the dependent variable and the more parameters the model has.
- MSE (mean squared error): the average of the squared prediction errors.
- AUC (area under the curve): the area under the ROC (receiver operating characteristics) curve that indicates the discriminatory capacity of the model.

Likewise, two techniques were used in order to compare the models:

- Repeated training test with different seeds (which consists of carrying out the process of partitioning and creating the model as many times as indicated, since with the repetition of the entire process all the data is used for creation and testing of the model and this reduces the overfit)
- Repeated cross-validation with different initialization seeds (the sample is divided into *k* subsets, where one of them is used as test data and the rest as training data; that is, it constructs the model with the data corresponding to *k* − 1 and evaluates with the rest. This process is repeated during *k* iterations and the result is the arithmetic mean of each one).

The analysis techniques used were: logistic regression, neural networks, random forest, gradient boosting and model assembly. The analysis techniques used are described in the next section.

## 4. Results

The study that has been carried out is of an analytical and observational type. It is analytical because it looked for the characteristics of the patients who returned to the hospital 72 h after discharge. In addition, it is observational because the study factor was not assigned by the researcher and was limited to observing, measuring and analyzing certain variables without exercising direct control of the intervention. Finally, from the temporal point of view, it is a cross-sectional and retrospective study that analyzed the data at a specific moment in the past.

Two programs from the SAS statistical processing package were used to perform the analysis.: SAS Enterprise Miner and SAS Base. The first program is interesting because of the large number of results it shows, including graphs and statistics even in the “black box” models, and the second program stands out for the method of evaluating the results (repeated cross-validation), which is more accurate than the one used with SAS Miner.

SAS Miner was used to create the logistic regression and neural network models, while the random forest models were built with SAS Base. This is because the SAS Miner Random Forest modeling node was not working properly and often gave errors. Gradient boosting and assembly models were carried out through the two programs, complementing some results with others. The idea of using both programs was the same: to take advantage of the main advantages of each to obtain more complete results.

### 4.1. Logistic Regression

This analysis used the imputed data. The processing was carried out as follows:

- In the first examination, a logistic regression was performed “forward,” “backward” and “step by step” for each of the sets of variables A, B and C. The results (Table 10) show that the misclassification rate was the same in all cases. Although the rest of the statistics vary between the models, nevertheless the best results were obtained with the backward selection method in all the sets of variables. The AUC value shows that the model with the highest discriminatory capacity was the one built with the set of variables C, followed by the models built with the set of variables B and A, in that order. Thus, it was necessary to do a new examination in order to determine which the dataset provided the optimal results.
- A training test of 10 repetitions and different seeds was carried out to determine the best set of variables. In each iteration, a logistic regression was performed with a backward selection method on each set of variables. The results (Table 11) show small differences between the statistics. However, the best values in terms of model quality throughout the iterations was obtained with the model built with the set of variables A (pathology2, reason_discharge2 and reason_discharge5).
- Finally, the global significance of the model (Table 12) was checked, obtaining a value of 0.0001 < 0.05.

### 4.2. Neural Network

**Figure 1:**
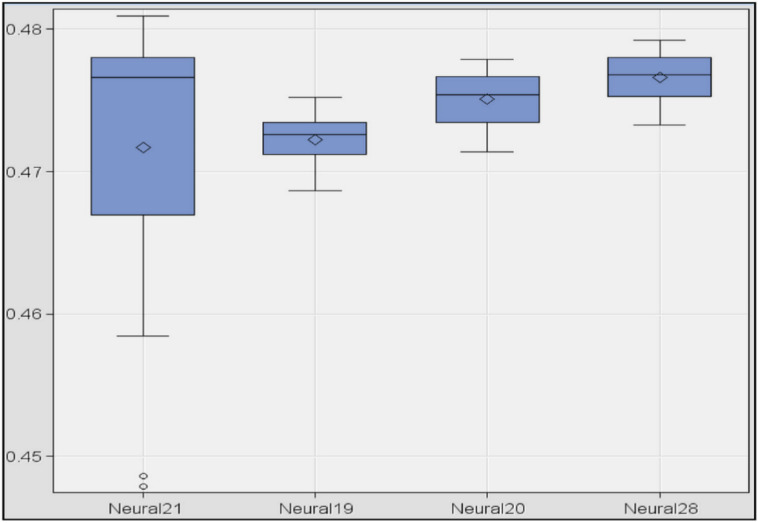
Box plot of the mean square error in neural network models.

The imputed dataset was used in this algorithm. To obtain the best model, the number of hidden layer nodes and the training algorithm were varied. Regarding the activation function, the function tanh (x) = 1 − (2/(1 + e^2x^) was always used as it works best. The processing was done as follows:

- First, four networks were built for each of the three sets of variables (12 network models). These four models consisted of the following parameters: three nodes and “bprop” algorithm, three nodes and “levmar” algorithm, seven nodes and “bprop” algorithm, and seven nodes and “levmar” algorithm. The result (Table 13) shows that the dataset C was the worst model since the AIC and SBC statistics were much higher than those of the other models, and the misclassification rate and the AUC were not able to be calculated. With respect of sets A and B, the misclassification rate was the same. However, the best values with respect to the AIC and the AUC were obtained by the set of variables A with seven nodes (regardless of the algorithm). However, the best SBC and MSE values were obtained by the set of variables A with three nodes (regardless of algorithm). Thus, the best set of variables was A. The optimal number of nodes will have to be studied since good results were achieved with three nodes and seven. Finally, it seems that the algorithm did not influence since the models were numerically the same with algorithm “levmar” and “bprop.”
- In the second exploration, six models were constructed using the set of variables A. Four of them were with the “levmar” algorithm and two of them with the “quasi-Newton” algorithm with the aim of checking whether the algorithm influenced the results. The influence of the number of nodes was also analyzed so that the four models with “levmar” consisted of 3, 5, 7 and 10 nodes, respectively, while the ”quasi-Newton” models had three and seven nodes. The result shows (Table 14) that the misclassification rate was similar in all models and that the algorithm did not influence the results, so the “levmar” algorithm was selected. In addition, it was observed that there was no model that was strictly better than the rest.
- In the third exploration, a training test was repeated 10 times with the four best models obtained: set of variables A, “levmar” algorithm and nodes 3, 7, 10 and 12, respectively. The result (Table 15) shows that the best models were those of three and seven nodes.
- Finally, in order to determine the best model, a box-plot diagram of the MSE of the models was done. The result shows (Figure 1) the diagrams for the models 10, 3, 7 and 12 nodes (in this order). The smallest errors appeared in the models with 10 and three nodes. However, the dispersion was greater in the first case. Comparing the models with three and seven nodes, it was observed that the model with three nodes had the smallest error, both in mean and variance. Therefore, the best model was obtained with the set of variables A, activation function “tanh,” algorithm “levmar” and a total of three nodes in the hidden layer.

### 4.3. Random Forest

In this algorithm, the dataset with missing values was used since it efficiently handled this type of data. Models were constructed by repeated cross-validation with various seeds in order to calculate the failure rate from the three sets of variables. The result is represented with a box-plot diagram.

**Figure 2:**
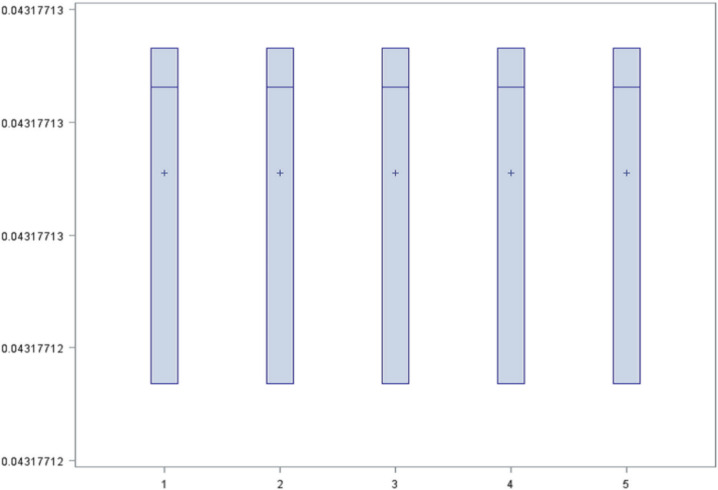
Box plot of the misclassification rate in random forest models.

For set A two models were created, one with 200 iterations and maximum depth of tree 50, and another with 50 iterations and maximum depth of tree 10. For set B, two models were created with the same parameters used for the set A. In addition, for set C only a single model was created (with 200 iterations and maximum depth of 50) due to the large number of variables. The diagram of result (Figure 2) shows that the rate had the same mean and distribution in all the models, therefore it was considered that all of them worked equally. For this reason, the simplest model was chosen as the best model. The model was the set of variables A (pathology2, reason_discharge2 and reason_discharge5) with 50 iterations, maximum depth of 10 and misclassification rate of 0.043.

### 4.4. Gradient Boosting

**Figure 3:**
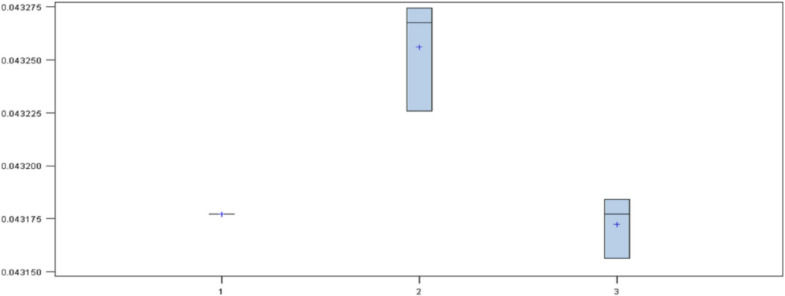
Box plot of the misclassification rate in gradient boosting models with sets of variables A, B and C.

**Figure 4:**
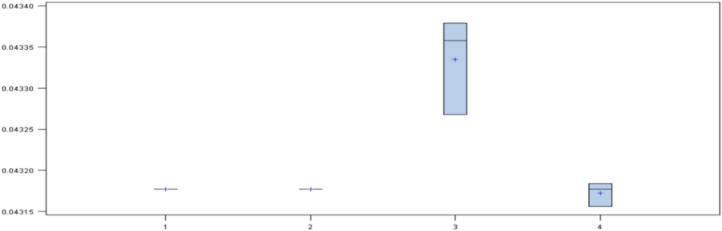
Box plot of the misclassification rate in gradient boosting models with sets of variables A and B.

**Figure 5:**
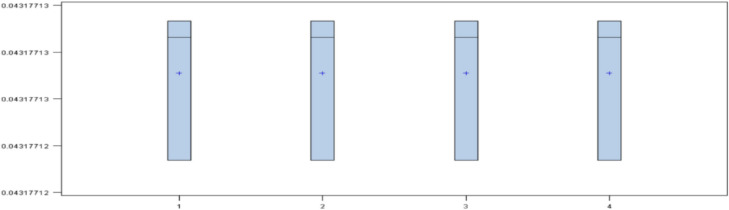
Box plot of the misclassification rate in gradient boosting models with sets of variables A.

In this algorithm, the dataset with missing values was used (since it efficiently handled this type of data) and the failure rate obtained is represented with a box-plot diagram. The processing was carried out as follows:

- First, a model was created for each dataset with the following configurations: for the set of variables A with 50 iterations a regularization constant of 0.2 was used; for the set B with 150 iterations a regularization constant of 0.1 was used; and for the set C with 250 iterations a regularization constant of 0.01 was used. The maximum depth of the three models was 2. The diagram (Figure 3) of results shows that the model with the highest mean was the set of variables B. The set of variables A and C had the same mean but different variance, with the set of variables A as the best of the three. Thus, the set of variables B was discarded.
- Next, four models were created: two with variables A (one with 50 iterations and a regularization constant of 0.2, and another with 250 iterations and regularization constant of 0.01), and the other two with variables C (in the same way). The diagram of the results (Figure 4) shows that there were three models with the same mean. However, the models of the set of variables A had the least variance, so the set of variables C was discarded.
- Next, four models were created with the set of variables A, varying the parameters corresponding to the iterations, the regularization constant and the maximum depth. The diagram of the results (Figure 5) shows that models were not influenced by the parameters, so the best model was the simplest.

Thus, the best model was obtained using the set of variables A (pathology2, reason_discharge2 and reason_discharge5) with parameters of 50 iterations, a regularization constant of 0.2 and maximum depth of 10.

### 4.5. Assemble

The assemblies were done using the best models obtained from each technique with missing and imputed datasets depending on each technique, assembling the techniques two by two, three by three and even four. The result shows (Table 16) that the misclassification rate and the AUC were the same in all cases, so the statistic to discriminate was the MSE. The lowest index was obtained with the assembly of regression and neural network followed by the assemblies of neural network and gradient boosting.

## 5. Discussions

The constructed models can be compared to analyze which best predicts the target variable. For this, Table 17 has been constructed which shows for each model studied (logistic regression, neural networks, random forest, and gradient boosting and model assembly) the misclassification rate, the MSE and the AUC.

The misclassification rate is the same for all models, so it is necessary to use the rest of the statistics to obtain a conclusion. If the AUC is considered, it is observed that the random forest and gradient boosting models should be discarded since they present the smallest values. Then, for the remaining models, the MSE is used to compare them. Therefore, it is observed that the model with the lowest value corresponds to the model of neural networks.

In order to validate this result, a cross validation was carried out, rebuilding the models and analyzing the results. In this sense, the same results were obtained. This may be due to two factors: an overfit is occurring or the sample is very homogeneous. To study if there is an overfit, the results obtained in the training and test sets were considered with the aim of verifying if the training results were very good (overfit) and if the test the results worsened significantly. Table 18 shows the results obtained and as it can be observed, there was no data overfit (the results are numerically very similar). Therefore, it can be concluded that the cause of the results being so similar in the models is because the dataset was very homogeneous.

The result of this research shows that the model that performs best is the one based on a neural network with activation function “tanh,” algorithm “levmar” and three nodes in the hidden layer. This is consistent with the results obtained by other authors previously cited in the introduction [36,37,39,42]. It also allows us to deduce that the phenomenon to be modeled corresponds to a non-linear model. This explains the best behavior of neural networks (they model nonlinear phenomena quite well). In this sense, it is very likely that the use of more advanced neural networks and a greater number of layers will allow us to obtain models that are closer to reality.

Another aspect of the research is the variables that explain the phenomenon. According to the results, the target variable could be explained based on the values of the dummy pathology2, reason_discharge2 and reason_discharge5 variables. The variable pathology2 is a dummy variable that represents whether a patient’s illness is related to general medicine. The reason_discharge2 variable is a dummy variable that represents the reason for the patient’s discharge from hospital. Finally, the variable reason_discharge5 is a dummy variable that represents the reason for the discharge of a patient that has left. In this sense, the result would indicate that the main reasons why a patient would be returning to the emergency department would depend on whether he has been treated for a general medicine problem or if the cause of discharge is due to hospitalization in the ward of the patient or to the evasion of the patient. Compared with the results of other studies [24], the results coincide with respect to the disease variable (although the same does not occur with the variables referred to medical discharge). Regarding the disease, other studies describe specific diseases [27,28,29] that influence return such as renal colic, spondiolysis or headache. In this sense, the result indicating “general medicine” diseases would be consistent, also taking into account that the possible groups that appear in the data (general medicine, traumatology, gynecology, ophthalmology, general pediatrics, obstetrics, pediatric trauma). It is very likely that patients with renal colic are classified in “general medicine,” just as in the case of headache. Therefore, the analysis carried out complements other works showing that in addition to the disease, another cause that influences return is the cause of the medical discharge.

Regarding the quality of the results, as shown, the differences between the models are minimal and the reason is not due to an overfit, but to the homogeneity of the samples. The explanation for this situation is due to the state of the data that has been used. Although the initial dataset has important dimensions (period between June 2015 and February 2018 with a total of 143,803 emergencies, of which 6209 returned in less than 72 h after discharge), they could not all be used for problems such as missing values or wrong data. For this reason, the results could be refined if any of the variables could be corrected. Likewise, variable selection methods could be improved using feature selection techniques.

## 6. Conclusions and Future Work

This article has carried out an analysis on the phenomenon of the return of patients to the emergency department of a hospital in less than 72 h. For this, the prediction of the target binary variable of the patient’s return has been studied using various machine learning algorithms with the aim of obtaining several models for the phenomenon studied and fixing the sets of variables that best explain it.

It has been verified that the neural network model with activation function “tanh,” algorithm “levmar” and three nodes in the hidden layer shows a better behavior than the rest of the studied models (since it presents a lower MSE than the rest of the models and a better AUC). In addition, the set of variables that best explain the phenomenon are pathology2 (corresponding to general medicine), reason_discharge2 (hospitalization of the plant) and reason_discharge5 (evasion). This result is consistent with what is stated in the studies of other authors about the non-linear nature of the phenomenon studied (since neural networks generally model phenomena that have non-linear behavior quite well).

On the other hand, it has been observed that the differences in the values of the statistics of the results obtained show very similar behaviors with the sets of variables used (this is because the results obtained are strongly influenced by the training sets and proof used). The explanation for this fact lies in the impossibility of having used all the variables available in the dataset due to the existence of erroneous data or missing data and the variable selection method used. This results in the fit of the data not being as good as it should be, and does not show a clearly winning model.

As future lines of work, several are proposed. First, analyze the sensitivity of the results according to the size of the source set. It may be valuable to other researchers and developers as it will provide them with a solid basis for determining the required size of the dataset. Second, the repetition of the study using feature selection techniques [42] to improve the selection of variables is needed. Third, use of other models of machine learning such as deep learning algorithms are needed since the results obtained in this study suggest that more sophisticated neural network models could better explain the studied phenomenon.

## Figures and Tables

**Table 1:** Descriptive analysis of the interval variables.

Name	Me	Miss	Total	Min	Max	σ	Skewness	Kurtosis
age	45	6	143,797	0	1849	24.88	2.63	190.76
blood_glucose	114	136,520	7283	40	600	79.43	2.70	8.44
cardiac_frequency	83	95,540	48,263	0	250	20.31	0.99	2.33
discharge_medical_time	157	0	143,803	−1356	18,010	319.441	9.71	197.28
discharge_min_time	160	0	143,803	−1356	18,010	332.719	9.05	172.70
emergency_min_time	1	140,455	3348	0	225	5.93	22.19	731.63
eve	6	83,148	60,655	0	10	1.79	−0.65	1.16
glasgow	15	136,918	6885	3	15	0.58	−14.16	228.59
new_triage	1	0	143,803	0	2	0.17	−5.22	27.41
observation_min_time	88	139,563	4240	−3	2283	212.318	2.73	12.06
press_arterial_max	136	100,221	43,582	53	270	25.17	0.51	0.61
press_arterial_min	80	100,259	43,544	0	150	15.25	0.12	0.75
query_min_time	41	2783	141,020	−36	8015	53.05	47.36	5.229.93
saturation_02	98	111,874	31,929	0	100	4.55	−8.85	136.15
short_treatment_min_time	154	134,094	9709	0	3287	264.983	2.36	7.80
temperature	37.1	123,664	20,139	33	41.5	1.03	0.67	0.14
triage_min_time	4	4003	139,800	−55	4143	12.57	265.96	85,255.86

**Table 2:** Description categorical variables.

Variable	Type	Levels	Missing
adequacy_consultation	C	5	142,730
bed_observation_area	N	24	139,563
bed_short_treatment	N	22	134,094
current_status_success	C	2	0
destination	C	25	25,190
emergency_revision	C	25	124,483
iccae_sn	C	1	139,863
incidents	C	1	125,221
interconsultation	C	1	143,063
level_triage	N	5	4388
medical_reconciliation	N	2	8349
pathology	C	7	4388
reason_discharge	C	15	0
reason_entry	C	16	0
sex	C	4	0
surgical_intervention	C	1	143,202
transfusions	C	1	142,892
transport	C	1	138,738
type_transport_income	C	5	738

**Table 3:** Variable interval modifications.

Variable	Old Value	New Value
age	0–1849	0–110
cardiac_frequency	0–250	30–230
discharge_medical_time	min = −1356	min = 0
discharge_min_time	min = −1356	min = 0
observation_min_time	min = −3	min = 0
query_min_time	min = −36	min = 0
time_triage_min	min = −55	min = 0

**Table 4:** Categorical Variable Modifications.

Variable	Old Categories	New Categories
destination	25 groups	4 groups: hospitalization, home, transfer and SHARE program
emergency_revision	25 groups	12 groups: rehabilitation, traumatology, ophthalmology, locomotor system, gynecology, ENT, internal medicine, cardiology, surgery, urology, digestive, pulmonology
iccae_sn	Yes / Empty	Empty category becomes “No”
incidents	Yes / Empty	Empty category becomes “No”
interconsultation	Yes / Empty	Empty category becomes “No”
pathology	7 groups	6 groups: general medicine, traumatology, gynecology, ophthalmology, general pediatrics, pediatric traumatology
reason_discharge	15 groups	7 groups: voluntary discharge, at home, to hospitalization, recovery or improvement, evasion, transfer and others
reason_entry	16 groups	5 groups: accident, health center referral, own initiative, others and emergencies
sex	4 groups	2 groups: F and M
surgical_intervention	Yes / Empty	Empty category becomes “No”
transfusions	Yes / Empty	Empty category becomes “No”
transport	Yes / Empty	Empty category becomes “No”
type_transport_income	5 groups	3 groups: own resources, ambulance and taxi

**Table 5:** Variable interval modifications.

Variable	Old Scale	New Scale
bed_observation_area	24 groups	Binary (1 = has occupied bed; 2 = has not occupied bed)
bed_short_treatment	22 groups	Binary (1 = has occupied bed; 2 = has not occupied bed)
return_72	7 groups	Binary (1,2,3,4,5,6 = 1; 0 = 0)

**Table 6:** Variable interval modifications.

Variable	Missing Values	Eliminated
adequacy_consultation	142,730	Yes
age	18	No
blood_glucose	136,520	Yes
cardiac_frequency	95,581	Yes
destination	25,190	No
discharge_medical_time	57	No
discharge_min_time	80	No
emergency_min_time	140,455	Yes
emergency_revision	124,483	Yes
eve	83,148	Yes
glasgow	136,918	Yes
level_triage	4388	No
medical_reconciliation	8349	No
observation_min_time	139,582	Yes
pathology	4388	No
press_arterial_max	100,221	Yes
press_arterial_min	100,259	Yes
query_min_time	2784	No
saturation_O2	11,874	Yes
sex	35	No
short_treatment_min_time	134,094	Yes
temperature	123,664	Yes
triage_min_time	4005	No
type_transport_income	738	No

**Table 7:** Selection of imputed data.

Method	Variables
R-square	level_triage2, level_triage4, level_triage5, pathology2 reason_discharge2, reason_discharge5
Partial least squares	pathology2, pathology5 reason_discharge2, reason_discharge5
“step by step” regression logistic	current_status_success, medical_reconciliation, sex, discharge_min_time, query_min_time, iccae_sn, incidents, level_triage2, level_triage3, level_triage5, surgical_intervention, transfusions, transport, type_transport_income2, pathology2, pathology5, pathology6, reason_entry3, destination3, destination5, reason_discharge2, reason_discharge3, reason_discharge5 reason_discharge6

**Table 8:** Selection of missing data.

Method	Variables
R-square	medical_reconciliation, level_triage2, level_triage3, transport, reason_discharge2, reason_discharge5, pathology2
Partial least squares	pathology2, reason_discharge2, reason_discharge5
Decision tree	discharge_medical_time, level_triage2, reason_entry2, reason_discharge5,

**Table 9:** Set of variables defined.

Dataset	Set A	Set B	Set C
Imputation	pathology2, reason_discharge2 and reason_discharge5	pathology2, pathology5, reason_discharge2, reason_discharge5, level_triage2 and level_triage5	Total variables: 50
Missing values	pathology2, reason_discharge2 and reason_discharge5	pathology2, reason_discharge2, reason_discharge5, level_triage2, reason_entry2, discharge_medical_time	Total variables: 50

**Table 10:** Initial comparison of logistic regression models.

Model	Misclassification Rate	AIC	SBC	MSE	AUC
Regression A “backward”	0.043	34,892.52	34,930.60	0.041	0.6
Regression A “forward”	0.043	35,811.95	35,821.47	0.041	0.5
Regression A “step by step”	0.043	35,811.95	35,821.47	0.041	0.5
Regression B “backward”	0.043	34,655.39	34,722.03	0.041	0.6
Regression B “forward”	0.043	35,811.95	35,821.47	0.041	0.5
Regression B “step by step”	0.043	35,811.95	35,821.47	0.041	0.5
Regression C “backward”	0.043	34,158.04	34,415.07	0.049	0.7
Regression C “forward”	0.043	35,811.95	35,821.47	0.041	0.5
Regression C “step by step”	0.043	35,811.95	35,821.47	0.041	0.5

**Table 11:** First iterations of training test of the best regression models.

Model	Misclassification Rate	AIC	SBC	MSE	AUC
Regression A-Iter 1	0.095	3363.50	3376.69	0.086	0.53
Regression B-Iter 1	0.095	3364.32	3377.50	0.086	0.52
Regression C-Iter 1	0.095	3364.32	3377.50	0.086	0.52
Regression A-Iter 2	0.059	1634.91	1644.34	0.055	0.52
Regression B-Iter 2	0.059	1635.53	1647.96	0.055	0.52
Regression C-Iter 2	0.059	1635.53	1647.96	0.055	0.52
Regression A-Iter 3	0.054	1542.78	1555.22	0.051	0.52
Regression B-Iter 3	0.054	1543.97	1562.63	0.051	0.52
Regression C-Iter 3	0.054	1543.97	1562.63	0.051	0.52
Regression A-Iter 4	0.053	1483.12	1495.50	0.050	0.52
Regression B-Iter 4	0.053	1484.76	1503.32	0.050	0.52
Regression C-ter 4	0.053	1484.76	1503.32	0.050	0.52

**Table 12:** Final regression model test.

Independent Terms Only	Independent Terms & Covariates	Likelihood Ratio Chi-Square	DF	Pr > ChiSq
35809.95	34884.52	925.43	3	<0.0001

**Table 13:** Initial comparison of neural network models.

Model	Misclassification Rate	AIC	SBC	MSE	AUC
NN.A.L3	0.043	318.68	509.07	0.40	0.5
NN.A.B3	0.043	318.68	509.07	0.40	0.5
NN.A.L7	0.043	225.82	644.68	0.41	0.6
NN.A.B7	0.043	225.82	644.68	0.41	0.6
NN.B.L3	0.043	336.68	612.75	0.40	0.5
NN.B.B3	0.043	336.68	612.75	0.40	0.5
NN.B.B7	0.043	408.68	1027.45	0.40	0.5
NN.B.L7	0.043	408.68	1027.45	0.40	0.5
NN.C.B3	-	979.58	2510.40	-	-
NN.C.L3	-	979.58	2510.40	-	-
NN.C.B7	-	974.87	4520.43	-	-
NN.C.L7	-	974.87	4520.43	-	-

**Table 14:** Second comparison of neural network models.

Model	Misclassification Rate	AIC	SBC	MSE	AUC
NN.A.L3	0.043	318.68	509.07	0.40	0.5
NN.A.L5	0.043	342.68	647.31	0.40	0.5
NN.A.L7	0.043	225.82	644.68	0.41	0.6
NN.A.L10	0.043	271.01	861.22	0.41	0.6
NN.A.Q3	0.043	318.68	509.07	0.40	0.5
NN.A.Q7	0.043	225.82	644.68	0.41	0.6

**Table 15:** First iterations of the training test of the best neural network models.

Model	Misclassification Rate	AIC	SBC	MSE	AUC
NN.A.L3-Iter1	0.041	49.85	173.61	0.42	0.5
NN.A.L7-Iter1	0.041	96.61	368.88	0.42	0.5
NN.A.L10-Iter1	0.041	132.76	516.43	0.41	0.5
NN.A.L12-Iter1	0.041	156.02	613.95	0.43	0.5
NN.A.L3-Iter2	0.043	49.62	172.67	0.42	0.5
NN.A.L7-Iter2	0.043	95.83	366.53	0.43	0.6
NN.A.L10-Iter2	0.043	131.95	513.38	0.43	0.5
NN.A.L12-Iter2	0.043	155.89	611.15	0.43	0.6
NN.A.L3-Iter3	0.047	49.81	172.94	0.42	0.5
NN.A.L7-Iter3	0.047	96.75	367.64	0.43	0.6
NN.A.L10-Iter3	0.047	131.36	513.06	0.43	0.5
NN.A.L12-Iter3	0.047	156.04	611.62	0.43	0.6
NN.A.L3-Iter4	0.044	49.84	173.26	0.42	0.5
NN.A.L7-Iter4	0.044	96.01	367.53	0.43	0.6
NN.A.L10-Iter4	0.044	132.10	514.71	0.43	0.5
NN.A.L12-Iter4	0.044	156.03	612.68	0.43	0.6

**Table 16:** Assembly results.

Model	Misclassification Rate	MSE	AUC
Reg+NN	0.043	0.040	0.61
NN+GB	0.043	0.041	0.61
Reg+GB	0.043	0.042	0.61
Reg+NN+GB	0.043	0.041	0.61
NN+Reg+GB+RF	0.043	NaN	0.6

**Table 17:** Model comparison.

Model	Misclassification Rate	MSE	AUC
Reg	0.043	0.041	0.61
NN	0.043	0.040	0.61
RF	0.043	0.042	0.50
GB	0.043	0.042	0.40
Reg+NN	0.043	0.041	0.61
NN+GB	0.043	0.041	0.61
Reg+GB	0.043	0.042	0.61
Reg+NN+GB	0.043	0.041	0.61

**Table 18:** Results for training and test sets.

Training			Test			
Model	Misclassification Rate	MSE	AUC	Misclassification Rate	MSE	AUC
Reg	0.043	0.041	0.61	0.043	0.041	0.61
NN	0.043	0.04	0.61	0.043	0.041	0.61
RF	0.043	0.04	0.62	0.043	0.041	0.6
GB	0.043	0.041	0.5	0.043	0.041	0.5
Reg+NN	0.043	0.04	0.61	0.043	0.041	0.61
NN+GB	0.043	0.041	0.61	0.043	0.041	0.61
Reg+GB	0.043	0.041	0.61	0.043	0.041	0.61
Reg+NN+GB	0.043	0.041	0.61	0.043	0.041	0.61

## Appendix A

The following tables describe the variables to be considered in the study, indicating their name, description, type and groups, if any. The type of a variable can be variables of type numeric, categorical or nominal free field.

**Table A1:** Nominal free field variables.

Name	Description
comment	Triage Nurse Free Text
clinical_judgment	Free field clinical opinion

**Table A2:** Integer variables.

Name	Description
admission_date	Date and time of the emergency admission
age	Age
blood_glucose	Blood glucose level collected in triage
cardiac_frequency	Heart rate
consultation_date	Date of consultation
discharge_date	Date and time of discharge
discharge_medical_date	Date and time of medical discharge (precedes “discharge_date”)
discharge_medical_time	Total time to discharge
discharge_min_time	Time until administrative discharge (occurs after “discharge_medical_time”)
emergency_date	Date and time of the patient’s entry to the emergency department
emergency_min_time	Time it takes for a patient to go into an emergency state
eve	Pain scale collected in triage
first_date_consultation	Date and time of the first medical attention
first_date_sol_lab	First date of request to the laboratory
first_date_sol_rad	First date of request to radiology
glasgow	Scale that assesses the level of consciousness, collected in triage
observation_min_time	Wait for admission under observation
new_triage	The system performs the triage again if the patient changes the clinical situation
press_arterial_max	Maximum blood pressure
press_arterial_min	Minimum blood pressure
query_min_time	Waiting for first consultation
reconsultation_last_year	Number of visits to the emergency department in the last year
saturation_O2	Oxygen saturation collected in triage
short_treatment_min_time	Wait until admission to the short treatment room
temperature	Temperature collected in triage
triage_date	Date and time recorded in triage room
triage_min_time	Waiting for triage

**Table A3:** Categorical variables.

Name	Description	Groups
adequacy_consultation	Adequacy consultation	5 groups: D1(adequate derivation), D2(reasonable derivation, but could have been avoided), D3(not suitable), IP1(it is appropriate that you have gone to the emergency room), IP3(unsuitable for having gone to the emergency room)
bed_observation_area	Concrete bed of the observation area	24 groups
bed_short_treatment	Concrete bed of the short treatment area	22 groups
current_status_success	Indicates if the patient has died	2 groups: Yes, No(Empty)
destination	Detailed description of the reason for discharge	25 groups
diagnostic_group	ICD-9 based diagnostic group	Many groups
diagnosis _main	Main diagnosis based on ICD-9	Many groups
doctor_discharge	Doctor discharging the patient	Many groups
doctor_family	Patient’s Family Physician	Many groups
emergency_revision	Referral to specialized consultation from the emergency department	25 groups
entity	Entity that insures the patient	Many groups
first_doctor_assigned	First doctor assigned	Many groups
first_doctor_consultation	First doctor with whom the consultation is held	Many groups
iccae_sn	It has a continuity of care report	2 groups: Yes, No(Empty)
incidents	Incidents	2 groups: Yes, No(Empty)
interconsultation	Interconsultation	2 groups: Yes, No(Empty)
level_triage	Triage level	5 groups:1(Emergency),2(non-delayed urgency),3 (Delay urgency),4(No urgency), 5(Administrative reason)
location	Location	Many groups
medical_reconciliation	It is assessed at discharge from the patient’s baseline treatment	3 groups: 0 (not reconciled), 1 (has been reconciled) or Empty (other cases).
nhc	Patient history number	---
pathology	Pathology	7 groups: General medicine, Traumatology, Gynecology, Ophthalmology, General, Pediatrics, Obstetrics, Pediatric trauma
procedures	Procedures performed based on the healthcare catalog	
processes	Assigned process based on healthcare catalog	Many groups
reason_consultation	Reason for the consultation (registered in triage)	Many groups
reason_discharge	Reason for discharge	15 groups
reason_entry	Reason for admission	16 groups
return_72	Number of consults after 72 h after discharge (target variable)	---
sex	Sex	4 groups: F(female),M(male), I(Indeterminate),U(unknown)
support_id	Unique identifier for each patient’s assistance (ID)	--
surgical_intervention	Surgical intervention	2 groups: Yes, No(Empty)
transfusions	Receive transfusion	2 groups: Yes, No(Empty)
transport	Go to the emergency department by transport	2 groups: Yes, No(Empty)
type_transport_income	Means of income transportation	5 groups: Own media, assisted ambulance, collective ambulance, individual ambulance, taxi
